# A Pool of Ferritin Nanoparticles Delivering Six Proteins of African Swine Fever Virus Induces Robust Humoral and Cellular Immune Responses in Pigs

**DOI:** 10.3390/vaccines14010093

**Published:** 2026-01-19

**Authors:** Zhanhao Lu, Dailang Zhong, Xin Song, Jing Lan, Yanjin Wang, Rui Luo, Shengmei Chen, Ruojia Huang, Hua-Ji Qiu, Yongfeng Li, Tao Wang, Yuan Sun

**Affiliations:** State Key Laboratory of Animal Disease Control and Prevention, National African Swine Fever Para-Reference Laboratory, National High Containment Facilities for Animal Diseases Control and Prevention, Harbin Veterinary Research Institute, Chinese Academy of Agricultural Sciences, Harbin 150069, China; luzhanhao106@163.com (Z.L.); hsrzlxcrb2021zdl@163.com (D.Z.); songxinor@163.com (X.S.); lanjing121@163.com (J.L.); wangyanjin1996@126.com (Y.W.); luorui20210423@163.com (R.L.); chenshengmei2023@163.com (S.C.); huangruojia2024@163.com (R.H.); qiuhuaji@caas.cn (H.-J.Q.); liyongfeng@caas.cn (Y.L.)

**Keywords:** African swine fever, African swine fever virus, antigen candidates, ferritin nanoparticle, immune responses

## Abstract

**Background/Objectives**: Inadequate characterization of protective antigens poses a significant challenge to the development of vaccines for African swine fever (ASF), particularly for antigen-dependent formulations such as subunit, mRNA, and recombinant viral vector vaccines. To address this, we aimed to screen African swine fever virus (ASFV) antigens and enhance their immunogenicity using a nanoparticle delivery platform. **Methods**: Here, six ASFV antigens (p30, p54, pE120R, pH124R, pE184L, and CD2v) were purified and used to immunize pigs individually. The effects of antibodies induced by these six antigens on ASFV replication or hemadsorption was evaluated in primary porcine alveolar macrophages (PAMs). These six antigens were, respectively, conjugated to ferritin via SpyTag/SpyCatcher to prepare six ferritin nanoparticles. A cocktail of the six mixed antigens or a cocktail of the six mixed nanoparticles was used to immunize pigs separately, and the differences in induced humoral and cellular immune responses were compared. **Results**: Antibodies generated against p30, p54, pE120R, pH124R, and pE184L in immunized pigs significantly inhibited ASFV replication in PAMs, while anti-CD2v antibodies specifically obstructed the hemadsorption of ASFV. Notably, immunization with a cocktail of these antigen-conjugated nanoparticles elicited a stronger virus-inhibitory antibody response compared to immunization with a cocktail of antigen monomers. Furthermore, nanoparticle immunization induced robust cellular immunity, evidenced by elevated serum IFN-*γ*, increased numbers of ASFV-specific IFN-*γ*-secreting cells, and an expanded CD8^+^ T cell population. **Conclusions**: Our study identifies a set of promising ASFV antigen candidates and demonstrates that ferritin nanoparticle delivery synergistically enhances both humoral and cellular immune responses against ASFV, providing a rational strategy for multi-antigen ASF vaccine design.

## 1. Introduction

African swine fever (ASF) is a highly contagious disease affecting domestic pigs and wild boars, with a mortality rate of up to 100%, which has led to substantial economic losses to the pig industry [1]. African swine fever virus (ASFV), which causes ASF, consists of 68 structural and over 100 non-structural proteins, nearly half of which have uncharacterized functions [2]. Various strategies have been explored for the development of ASF vaccines. Inactivated vaccines fail to confer protection against ASF [3]. Live attenuated vaccines (LAVs) can offer homologous protection and even confer cross-protection. However, they may exhibit residual virulence and reversion to virulence, alongside the risk of recombination [4,5,6]. Viral vectored vaccines provide partial protection, but subclinical symptoms such as viremia and fever may persist [7]. Subunit vaccines have achieved only limited protection, primarily due to insufficient understanding of protective antigens and challenges in selecting an effective delivery system [8,9,10]. Currently, the development of a safe and effective ASF vaccine remains elusive.

The ASFV virion possesses a five-layer structure, which harbors numerous candidate antigens for vaccine development [11,12]. CD2v, an outer envelope protein of ASFV, is a key molecule that promotes viral spread within the host through hemadsorption, and its extracellular domain contains multiple epitopes valuable for vaccine design [13,14]. Antibodies targeting p30 and the inner envelope protein p54 can inhibit ASFV internalization and attachment, respectively, and combining immunization with these two antigens provides partial protection against ASFV challenge, making them highly promising target antigens [15,16]. pE120R is located in the viral capsid and is closely associated with virus release [17]. Non-structural protein pE184L interacts with STING to block interferon production for viral replication and pathogenesis [18]. Immunization of pigs with a combination of four proteins (p30, p54, p72, and p22) or with a combination of CD2v, p30, and pK205R only delayed the onset of clinical symptoms [19,20]. Therefore, vaccines developed based on these known immunogens cannot provide satisfactory protection. A recent proteome-wide study of antibody dynamics suggests that structural proteins like pE120R and non-structural proteins such as pE184L and pB602L may also be excellent candidates for vaccines [21]. However, research on the screening and application of these potential novel antigens remains insufficient.

Protein nanoparticles are self-assembled biomolecular structures that can stably display antigens on their surface, closely mimicking the structure of natural viral particles [22]. These nanoparticles have been shown to enhance the uptake by immune cells, thereby improving antigen presentation and eliciting robust antibody responses along with potent cellular immunity [23,24]. Among various nanoplatforms, ferritin-based nanoparticles exhibit significant potential for inducing protective immunity against a range of viral pathogens [25,26,27]. Ferritin is a naturally occurring, non-toxic, and thermally stable protein that can be produced at scale using simple, rapid, and cost-effective methods [28]. Additionally, the SpyTag/SpyCatcher coupling system allows convenient and efficient conjugation of antigens onto the nanoparticle surface, facilitating the straightforward and rapid assembly of recombinant antigen-displaying nanoparticles [29].

In this study, we selected six antigens—p30, p54, pE120R, pH124R, pE184L, and CD2v. Antibodies of anti-p30, p54, pE120R, pH124R, and pE184L exhibited inhibitory effects against ASFV in porcine alveolar macrophages (PAMs), while antibodies against CD2v significantly reduced ASFV hemadsorption. Moreover, pigs immunized with a mixture of six antigen-conjugated ferritin nanoparticles utilizing the SpyTag/SpyCatcher demonstrated significantly enhanced ASFV inhibitory effects compared with those receiving mixed antigen monomers immunization, achieving an inhibition rate of 70.9%, akin to that observed in sera from convalescent pigs. In terms of cellular immunity, immunization with antigen-conjugated nanoparticles elicited elevated levels of ASFV-specific IFN-*γ*-secreting effector cells and CD8^+^ T cells, along with increased serum IFN-*γ* levels. Our findings provide valuable insights for identifying antigen candidates and selecting delivery systems for the future development of ASF subunit vaccines.

## 2. Materials and Methods

### 2.1. Cells and Viruses

Primary porcine alveolar macrophages (PAMs) were isolated from the lung lavage fluid of 28-day-old healthy specific pathogen-free (SPF) piglets that were purchased from Experimental Animal Base of Harbin Veterinary Research Institute (HVRI) of Chinese Academy of Agricultural Sciences (CAAS), and cultured in RPMI 1640 medium with L-glutamine (Thermo Fisher Scientific, Waltham, MA, USA). The medium was supplemented with antibiotics (100 U/mL of penicillin and 100 mg/mL streptomycin, Gibco, Carlsbad, CA, USA) and 10% (*v*/*v*) fetal bovine serum (FBS) (Gibco). The Expi-CHO-S cell line (Thermo Fisher Scientific, Waltham, MA, USA) was cultured in CHOGrow CD2 serum-free medium (H120KJ, BesalMedia, Shanghai, China) supplemented with L-glutamine. Human embryonic kidney-derived HEK293T cells (Procell, Wuhan, China) were grown in Dulbecco’s modified Eagle’s medium (DMEM) (Gibco, Carlsbad, CA, USA) supplemented with 10% FBS. The ASFV HLJ/18 strain (ASFV-WT) (GenBank accession number MK333180.1) was isolated as previously described [30]. The recombinant reporter ASFV (rASFV-Gluc/EGFP) has been described previously, which enables in vitro identification of infected cells and quantification of ASFV replication [31].

### 2.2. Construction of Plasmids

Six ASFV antigens were used in this study: p30 (*CP204L*), p54 (*E183L*), pE120R, pH124R, pE184L, and CD2v. The gene sequences for all antigens were derived from the ASFV-WT. The extracellular domains were retained for p54 (amino acids 53–184) and CD2v (amino acids 1–196). The plasmids pUC-CP204L, pUC-E183L, pUC-E120R, pUC-H124R, and pUC-E184L, synthesized by Shanghai Sangon Biotech (Shanghai, China), were fused to the *ferritin* gene (UniProt: Q9ZLI1) using primers Ag-F/R and ferritin-F/R, resulting in *CP204L-ferritin*, *E183L-ferritin*, *E120R-ferritin*, *H124R-ferritin*, and *E184L-ferritin*, respectively. The fusion genes were cloned into the pCold-I vector to generate pCold-CP204L-ferritin, pCold-E183L-ferritin, pCold-E120R-ferritin, pCold-H124R-ferritin, and pCold-E184L-ferritin, respectively. Recombinant plasmids pCold-CP204L-ST, pCold-E183L-ST, pCold-E120R-ST, pCold-H124R-ST, and pCold-E184L-ST were constructed using PCR-based site-directed mutagenesis with the primers SpyTag (ST)-F/R. The nucleotide sequences of all inserts in these plasmids were verified using Sanger DNA sequencing. The plasmid pCold-SpyCatcher-ferritin expressing SpyCatcher-ferritin (SC-ferritin) has been described previously [32]. The eukaryotic expression plasmids for the six antigens (pCAGGS-CP204L, pCAGGS-E183L, pCAGGS-E120R, pCAGGS-H124R, pCAGGS-E184L, and pCAGGS-EP402R) were stored in our laboratory. The plasmid pLVX-EP402R was stored in our laboratory. Primers used in this study are listed in Table 1.

### 2.3. Expression of ASFV Antigen Candidates and Production of Nanoparticles

The CHO-EP402R-SpyTag cell line was constructed following established protocols described previously [32]. CD2v-SpyTag (CD2v-ST) protein was continuously secreted and expressed by the cell line. Upon reaching a cell density of 10^8^ cells/mL in suspension culture, the supernatant was harvested by centrifugation. The supernatant was then filtered using a 0.45-µm Millex HV filter (SLHV033R, Merck Millipore, Billerica, MA, USA) to remove any particles or debris. Subsequently, the filtered supernatant was incubated with Ni Sepharose High-Performance resin (17526802, Cytiva, Marlborough, MA, USA) for affinity purification of the CD2v-ST protein.

p30-ST, p54-ST, pE120R-ST, pH124R-ST, and pE184L-ST were expressed in *Escherichia coli*. The plasmids pCold-CP204L-ST, pCold-E183L-ST, pCold-E120R-ST, pCold-H124R-ST, and pCold-E184L-ST were transformed into BL21 (DE3) competent cells (Tiangen, Beijing, China), respectively. Single clones of transformed cells were cultured in LB medium supplemented with 100 μg/mL ampicillin at 37 °C with shaking (200 rpm). When the OD_600nm_ of the culture reached approximately 0.6, isopropyl-*β*-D-thiogalactopyranoside (TaKaRa, Tokyo, Japan) was added to a final concentration of 1 mM to induce protein expression. The culture was incubated at 16 °C for 18 h, after which the bacterial cells were harvested by centrifugation and lysed by sonication. Following lysis, the bacterial lysate was centrifuged, and the supernatant was incubated with Ni-NTA resin (Cytiva, USA) to enrich the His-tagged proteins pE120R-ST, pH124R-ST, pE184L-ST, and SC-ferritin. Subsequently, the proteins were eluted with imidazole in Tris (hydroxymethyl) aminomethane hydrochloride (Tris-HCl) buffer. The purified proteins were then concentrated and buffer-exchanged with phosphate-buffered saline (PBS) instead of Tris-HCl buffer. The expression and characterization of SC-ferritin have been previously described [32].

The monomeric antigens p30-ST, p54-ST, pE120R-ST, pH124R-ST, pE184L-ST, and CD2v-ST were individually conjugated to SC-ferritin via stable covalent bonds to obtain the six types of nanoparticles, p30-ferritin (Fer-p30), p54-ferritin (Fer-p54), pE120R-ferritin (Fer-E120R), pH124R-ferritin (Fer-H124R), pE184L-ferritin (Fer-E184L), and CD2v-ferritin (Fer-CD2v). Purified SC-ferritin was incubated with each monomeric antigen, respectively, overnight at 4 °C in 50 mM PBS (pH 7.3). The resulting conjugates were purified using a Superose 6 Increase 10/300 GL gel filtration column pre-equilibrated with 50 mM 4-(2-Hydroxyethyl) piperazine-1-ethanesulfonic acid (HEPES) (pH 7.3) and 300 mM NaCl. The eluted fractions were analyzed and stored at 4 °C.

### 2.4. SDS-PAGE and Western Blotting

Equal amounts of each recombinant protein were analyzed using 12.5% SDS-PAGE. The gel was stained with Coomassie Brilliant Blue or transferred to polyvinylidene difluoride membranes (Sigma-Aldrich, St. Louis, MO, USA). The membranes were blocked with 5% skim milk for 2 h at room temperature, followed by overnight incubation with either an anti-His monoclonal antibody (mAb) (1:5000 dilution, Sigma) or swine anti-ASFV sera (1:200 dilution) at 4 °C. After washing five times with 0.05% Tween-20 in PBS (PBST), the membranes were incubated for 1 h at room temperature with horseradish peroxidase (HRP)-conjugated goat anti-mouse IgG (1:5000 dilution, Abcam, Cambridge, MA, USA) or HRP-conjugated goat anti-pig IgG (1:5000 dilution, Abcam). After washing with PBST, the blots were visualized using an enhanced chemiluminescence reagent (Thermo Fisher Scientific, Waltham, MA, USA).

### 2.5. Indirect Immunofluorescence Assay (IFA)

HEK293T cells were transfected with pCAGGS-CP204L, pCAGGS-E183L, pCAGGS-E120R, pCAGGS-H124R, pCAGGS-E184L, and pCAGGS-EP402R, respectively. At 36 h post-transfection, the cells were fixed with absolute ethanol at −30 °C for 20 min, followed by two washes with PBS. The fixed cells were incubated with immunized pig sera diluted by 1:10 at 37 °C for 2 h. After washing with PBS, the cells were incubated with a FITC-conjugated anti-porcine IgG secondary antibody (diluted 1:100) at 37 °C for 1 h. The secondary antibody solution was discarded, and the cells were resuspended in 200 μL of PBS for analysis of antibody binding using an inverted fluorescence microscope.

### 2.6. Indirect Enzyme-Linked Immunosorbent Assay (iELISA)

The optimal coating concentrations for recombinant proteins p30, p54, pE120R, pH124R, pE184L, and CD2v were determined by checkerboard titration. Under these conditions, the cut-off values (average ± 5 times the standard deviation) were determined using 50 ASFV-negative pig sera obtain from a pig farm in Heilongjiang Province, China (Table S1). Antigen-specific IgG responses in the sera of vaccinated pigs were measured using in-house iELISAs. Briefly, 96-well microtiter plates were coated with recombinant antigens overnight at 4 °C and blocked with 5% skim milk for 2 h at 37 °C. Serum samples (1:100 dilution) from immunized pigs were added to each well and incubated for 1 h at 37 °C. After five washes with PBST, the plates were incubated with HRP-conjugated anti-pig IgG (1:10,000 dilution) for 1 h at 37 °C. After washing, the color was developed with 3,3′,5,5′-tetramethylbenzidine substrate for 15 min at 37 °C and stopped by 2 M H_2_SO_4_. The OD_450nm_ value of each well was determined using a Bio-Tek Epoch2 microplate reader.

### 2.7. Virus Inhibition Assay in PAMs

PAMs were seeded in 96-well cell culture plates at a density of 10^5^ cells/well. Prior to antibody function assay, all sera were heat-inactivated at 56 °C for 30 min. Ten-fold diluted inactivated sera were mixed with rASFV-Gluc/EGFP (MOI = 0.1). After incubation for 1.5 h at 37 °C, 100 µL of the mixture was added to each well, and the plates were incubated at 37 °C for 72 h. SPF pig sera and sera from convalescent pigs immunized with ASFV-SY18-∆CD2v/UK served as the negative and positive controls, respectively [33]. The Gluc activities in the supernatants were detected as previously described [31].

### 2.8. Hemadsorption Inhibition Assay (HADIA)

PAMs were seeded into 96-well cell culture plates at a density of 10^4^ cells/well and cultured at 37 °C for 12 h. The cells were then infected with ASFV-WT at an MOI of 0.1. At 24 h post-infection (hpi), the ASFV-WT-infected PAMs were assessed. Anti-CD2v sera were heat-inactivated at 56 °C for 30 min and incubated with ASFV-WT-infected PAMs at a dilution of 1:10 for 2.5 h. Subsequently, 1% swine red blood cells (RBCs) were added, and plates were incubated for an additional 36 h at 37 °C. Hemadsorption (rosette formation) was then observed under a microscope.

### 2.9. Size-Exclusion Chromatography (SEC)

The recombinant proteins p30-ST, p54-ST, pE120R-ST, pH124R-ST, pE184L-ST, and CD2v-ST were individually incubated with SC-ferritin in conventional HEPES for 24 h, respectively. The resulting ST/SC conjugated proteins were purified using SEC as described previously [32].

### 2.10. Transmission Electron Microscope (TEM)

Approximately 5 μL aliquots of the purified nanoparticles, at concentrations ranging from 0.05 to 2 mg/mL, were applied to freshly glow-discharged 300-mesh copper grids and incubated for 1 min. The grids were washed twice with double-distilled water, blotted, and negatively stained with freshly prepared 2% (*w*/*v*) uranyl acetate for 45 s before being air-dried. The grids were imaged using an H-7650 transmission electron microscope.

### 2.11. Pig Vaccination Experiments

Experiment 1

Eighteen four-week-old ASFV-free piglets were included to assess the immunogenicity of ASFV antigens, and the detection method is described previously [34]. The piglets were randomly assigned to six groups of three pigs each and housed under identical conditions with unrestricted access to food and water. Following a 24 h acclimation period, pigs were vaccinated with purified recombinant proteins p30-ST, p54-ST, pE120R-ST, pH124R-ST, pE184L-ST, and CD2v-ST. A dose of 100 µg of each antigen was diluted in 1 mL of PBS with 15% adjuvant MONTANIDE ISA 15A VG, injected intramuscularly, and boosted two and four weeks later. Sera were collected at 0, 14, 28, and 42 days post-vaccination (dpv) to assess the immunogenicity. All piglets were euthanized via intravenous injection with sodium pentobarbital (Bio PIKE, Beijing, China) at 42 dpv.

Experiment 2

Eleven four-week-old ASFV-free piglets were included to evaluate the immunogenicity of ASFV antigen combinations. All pigs were randomly assigned to three groups randomly and housed under identical conditions with unrestricted access to food and water. Following a three-day acclimation period, Group A consisted of 3 pigs, each immunized with PBS as a negative control. Group B consisted of 4 pigs, each immunized with a mixture of six soluble monomeric antigens (100 µg each of p30-ST, p54-ST, pE120R-ST, pH124R-ST, pE184L-ST, and CD2v-ST, totaling 600 µg). Group C consisted of 4 pigs, each immunized with a mixture of six nanoparticles (Fer-p30, Fer-p54, Fer-E120R, Fer-H124R, Fer-E184L, and Fer-CD2v; each nanoparticle delivered 100 µg of its respective antigen, totaling 600 µg of effective antigen). An amount of 1 mL of the containing six antigen monomers or six nanoparticle formulations was emulsified with MONTANIDE ISA 15A VG adjuvant and administered via intramuscular injection for immunization in pigs. Booster vaccinations were subsequently administered at two and four weeks following the initial inoculation. Sera and peripheral blood mononuclear cells (PBMCs) were collected at 0, 14, 28, and 42 dpv to assess immune responses.

### 2.12. Enzyme-Linked Immunosorbent Assay (ELISA)

The concentrations of TNF-*α* (ELP-TNFa-1, RayBiotech, Norcross, GA, USA) and IFN-*α* (ELP-IFNa-1, RayBiotech, Norcross, GA, USA) in the serum were measured by enzyme-linked immunosorbent assay (ELISA) kits according to the manufacturer’s instructions. The IFN-*γ* concentrations in sera at 0, 14, 28, and 42 dpv after immunization were measured using a double-antibody sandwich ELISA kit (ml002333, MLBio, Shanghai, China) following the manufacturer’s instructions.

### 2.13. Enzyme-Linked Immunosorbent Spot (ELIspot)

To assess the ASFV-specific cellular immune responses, an ELIspot assay was performed using a porcine IFN-*γ* ELIspot kit (MBT-3130-4HPW-2, Mabtech, Nacka, Sweden). Briefly, PBMCs from peripheral blood treated with heparin (Solarbio, Beijing, China) were collected at 42 dpv and adjusted to a concentration of 10^6^ cells/mL using RPMI 1640 medium containing 10% FBS for 20 h. PBMCs were stimulated with the ASFV-WT (10^6.0^ TCID_50_). RPMI 1640 medium and phytohemagglutinin served as the negative and positive controls, respectively. After 20 h of stimulation, spot-forming units were quantified using an iSpot Spectrum ELR088IFL reader as described previously [35].

### 2.14. Flow Cytometry

Around 10^6^ PBMCs were isolated from each immunized pig and divided into five groups. One group was incubated with anti-CD3, -CD4, and -CD8 antibodies (4510-13, 4515-02, 4520-09, SouthernBiotech, Birmingham, AL, USA) at 4 °C in the dark for 30 min. The untreated group and three single-staining groups were incubated with anti-CD3, -CD4, and -CD8 antibodies, respectively, to trap and regulate fluorescence compensation. Stained PBMCs were washed with PBS and resuspended after centrifugation. The cells were resuspended in 0.5 mL of PBS and filtered into a tube for flow cytometry. Cytometry assays were conducted using Cytomics FC 500.

### 2.15. Statistical Analysis

Student’s *t*-test was used to assess the statistical significance of differences in virus inhibition assay and HADIA results between immune sera collected at 0 and 42 dpv from Experiment 1. One-way ANOVA was employed to analyze the differences in cytokines TNF-*α* and IFN-*α* concentration in 14 dpv sera, antibody titers and virus inhibition rates in 42 dpv sera, IFN-*γ*-secreting effector cell numbers and CD4^+^/CD8^+^ T cell percentages in 42 dpv PBMCs, and the changes in virus inhibitory effects during the immune process (0, 14, 28, and 42 dpv) of each group among groups A, B, and C from Experiment 2. Two-way ANOVA was used to analyze the differences in IFN-*γ* concentrations among groups A, B, and C during the immune process (0, 14, 28, and 42 dpv). *p* ≥ 0.05 was considered statistically non-significant (ns), *p* < 0.05 was considered statistically significant (* *p* < 0.05, ** *p* < 0.01, *** *p* < 0.001). GraphPad Prism Version 8.0.0 (GraphPad Software, San Diego, CA, USA) was used to construct data graphs GraphPad Prism version.

## 3. Results

### 3.1. Selection, Design, and Validation of ASFV Antigen Candidates

**Figure 1:**
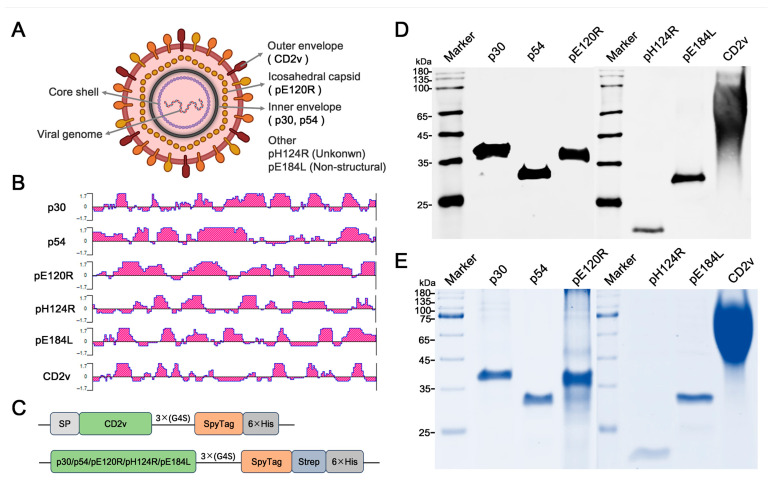
Selection, design, and validation of ASFV antigen candidates. (**A**) Localization of antigen candidates within the ASFV virion. Each layer of the viral particle is shown in distinctive colors, including the outer envelope, icosahedral capsid, inner envelope, core–shell, viral genome, and other components. Antigens selected in this study are depicted under each layer. (**B**) Prediction of the immunodominant epitopes of the antigen candidates. The most antigenic regions of p30, p54, pE120R, pH124R, pE184L, and CD2v were predicted by the Protean program (DNASTAR, Madison, USA) using the GenBank reference sequence (GenBank No. MK333180.1). (**C**) Design for expression strategies of the antigen candidates SP: signal peptide. 3 × G4S: the linker. SpyTag: a part of the coupling system. 6 × His: a tag used for protein purification. Strep: another tag for protein purification. (**D**,**E**) Validation of the purified recombinant proteins. Western blotting (**Upper**) and Coomassie Brilliant Blue staining (**Lower**) analysis of p30-ST, p54-ST, pE120R-ST, pH124R-ST, pE184L-ST, and CD2v-ST.

p30, p54, and CD2v have been identified as ASFV potential antigen candidates [15,16,20,36] (Figure 1A). According to the results predicted by DNASTAR, p30, p54, and CD2v contained multiple epitopes with an antigen index of 1.7, indicating that they may have good immunogenicity (Figure 1B). Additionally, the structural protein pE120R and non-structural proteins pH124R and pE184L demonstrated significant reactogenicity with the convalescent swine sera in Western blotting [37] and predicted to have the same level of immunogenicity (Figure 1A,B). Consequently, these six antigens were included in the antigen pool for validating antigen candidates. Different strategies were designed for the protein expression of recombinant antigens (Figure 1C and Figure S1). After exploring the conditions for antigen purification, six recombinant antigens were obtained (Figure S2). The results of Western blotting and Coomassie Brilliant Blue staining showed that the purified recombinant antigens were specific and uniform and could be used for further immunogenicity assays in pigs (Figure 1D,E).

### 3.2. Evaluation of the Immunogenicity of the Antigen Candidates in Pigs

**Figure 2:**
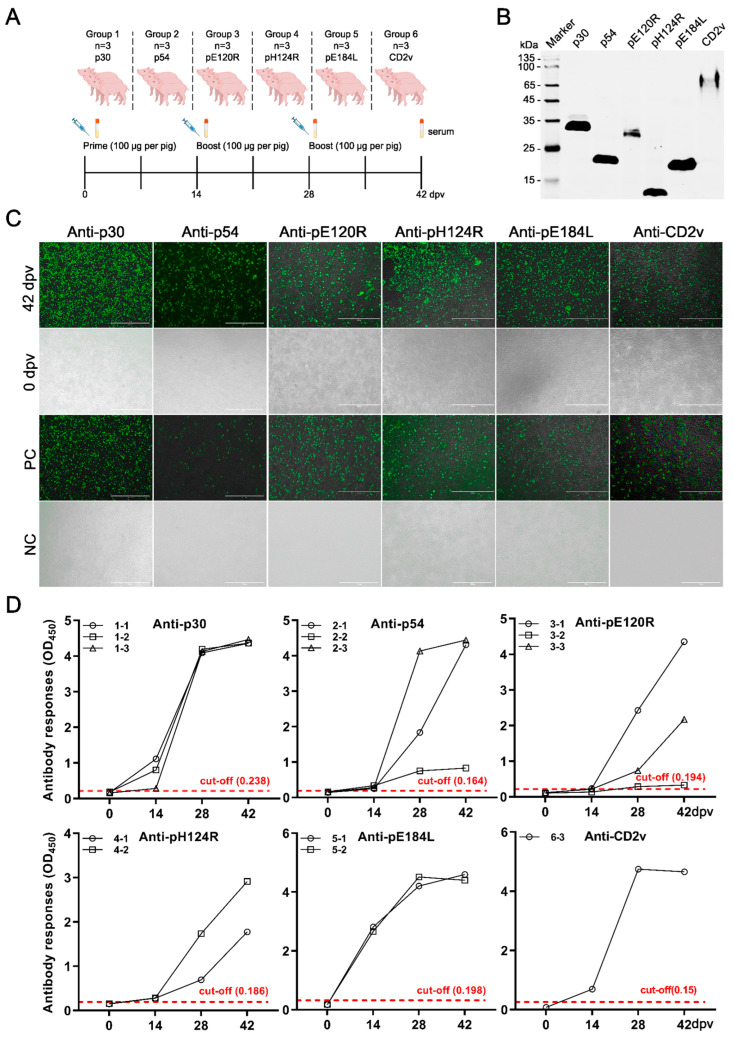
Evaluation of the immunogenicity of the antigen candidates in pigs. (**A**) Schematic diagram of pig vaccination. Each pig was vaccinated with 100 μg of antigens at days 0, 14, and 28. Blood was collected at 0, 14, 28, and 42 days post-vaccination (dpv). (**B**) Verification of eukaryotic expression plasmids by Indirect immunofluorescence assay (IFA). HEK293T cells were transfected with plasmid pCAGGS-p30, pCAGGS-p54, pCAGGS-pE120R, pCAGGS-pH124R, pCAGGS-pE184L or pCAGGS-CD2v. At 24 h post-transfection, the cells were lysed with RIPA buffer and the whole cell lysates were verified by Western blotting with an anti-Flag monoclonal antibody. (**C**) IFA for ASFV antigen-specific antibody detections. By detecting the sera at 42 dpv to determine whether the antibody turned positive. Sera collected at 0 dpv were the negative control, and anti-Flag antibody was used as the positive control. (**D**) Antigen-specific antibody titers were determined by in-house indirect enzyme-linked immunosorbent assay (iELISA) for p30, p54, pE120R, pH124R, pE184L, and CD2v at 0 and 42 dpv. The dashed lines represent the cut-off value for each iELISA. Group 1: pigs vaccinated with p30 (1-1, 1-2 and 1-3; *n* = 3); Group 2: pigs vaccinated with p54 (2-1, 2-2 and 2-3; *n* = 3); Group 3: pigs vaccinated with pE120R (3-1, 3-2 and 3-3; *n* = 3); Group 4: pigs vaccinated with pH124R (4-1 and 4-2; *n* = 2); Group 5: pigs vaccinated with pE184L (5-1 and 5-2; *n* = 2); Group 6: pigs vaccinated with CD2v (6-3; *n* = 1). The reduced sample sizes in Groups 4-6 were due to unintended infection, and data from these groups should be interpreted as indicating immunological trends rather than definitive outcomes.

Antigen immunogenicity was determined by detecting the antibody response. Pigs were immunized at 0, 14, and 28 dpv, respectively (Figure 2A). The six eukaryotic expression plasmids were verified by Western blotting and used for the detection of serum antibodies by IFA (Figure 2B). The IFA results showed that the antibodies at 42 dpv were positive in contrast to the sera at 0 dpv (Figure 2C and Figure S3). To analyze antibody levels after immunization, we developed six in-house iELISA methods for each antigen. The sera of all pigs were detected under the premise that the OD_450nm_ of all sera before immunization was lower than the corresponding cut-off value, whereas sera from the p30, p54, and pE120R immunization groups, as well as from the smaller groups immunized with pH124R, pE184L, and CD2v, displayed a trend of increasing OD_450nm_ values at 14, 28, and 42 dpv compared to 0 dpv (Figure 2D). These results indicated that the six antigens were highly immunogenic.

### 3.3. Identification of ASFV Antigen Candidates

**Figure 3:**
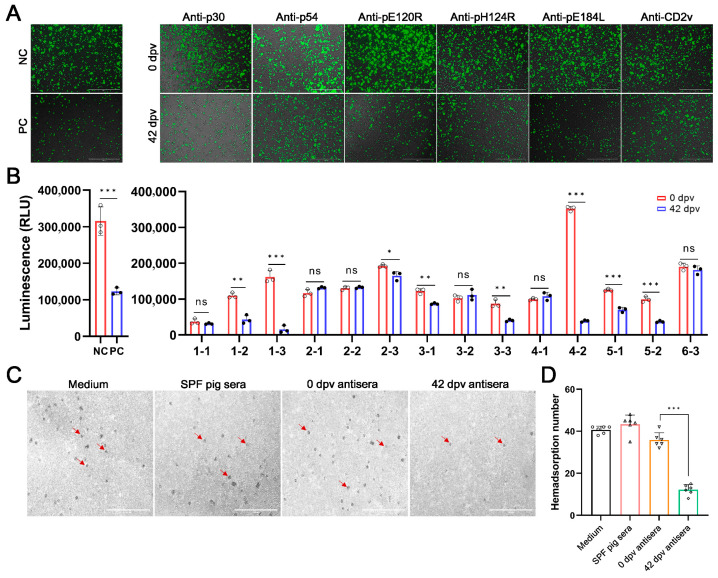
Identification of ASFV antigen candidates. (**A**) Fluorescence observation for inhibition of rASFV-Gluc/EGFP replication by ASFV antigen-specific antibodies. After treatment with serum collected at 0 or 42 dpv from each pig, Primary porcine alveolar macrophages (PAMs) infected with rASFV-Gluc/EGFP were observed via fluorescence microscopy at 72 h post-infection (hpi). SPF pig sera served as the negative control (NC), and the sera of convalescent pigs surviving ASFV infection were used as the positive control (PC). (**B**) Gluc activities analysis for rASFV-Gluc/EGFP inhibition by ASFV antigen-specific antibodies. At 72 hpi, the PAMs culture supernatants were collected to detect Gluc activities, and the inhibition effects of the antibodies were analyzed. NC and PC are the same as (**A**). (**C**) Hemadsorption inhibition of CD2v antisera. PAMs were infected with ASFV-WT at a multiplicity of infection of 0.1. RPMI 1640 medium, SPF pig sera, or CD2v antisera was incubated at 24 hpi. Hemadsorption was observed after adding 30 μL of 1% red blood cells for 36 h. (**D**) Quantitative analysis for hemadsorption. Six random fields were statistically analyzed. Group 1: pigs vaccinated with p30 (1-1, 1-2 and 1-3; *n* = 3); Group 2: pigs vaccinated with p54 (2-1, 2-2 and 2-3; *n* = 3); Group 3: pigs vaccinated with pE120R (3-1, 3-2 and 3-3; *n* = 3); Group 4: pigs vaccinated with pH124R (4-1 and 4-2; *n* = 2); Group 5: pigs vaccinated with pE184L (5-1 and 5-2; *n* = 2); Group 6: pigs vaccinated with CD2v (6-3; *n* = 1). The data were analyzed using the Student’s t-test (**B**) or one-way ANOVA (**D**), bars represent the means ± SD; ns, not significant (*p* > 0.05); *, *p* < 0.05; **, *p* < 0.01; ***, *p* < 0.001. For antigens pH124R, pE184L, and CD2v, functional assays were performed with sera from a reduced number of animals (Group 4, *n* = 2; Group 5, *n* = 2; Group 6, *n* = 1; due to unintended infection). Data from these groups are presented to show indicative trends.

To further verify whether the antibodies against ASFV antigens had an inhibitory, promoting, or no effect on ASFV replication, we first used rASFV-Gluc/EGFP for evaluation. EGFP of rASFV-Gluc/EGFP causes the infected PAMs to emit green fluorescence. Gluc expressed by rASFV-Gluc/EGFP catalyzes substrate luminescence, and light intensity is linearly related to the number of viruses [31]. Under fluorescence microscopy, PAMs treated with SPF pig sera exhibited bright green fluorescence. In contrast, a significant attenuation of fluorescence was observed in PAMs incubated with convalescent sera, indicating effective inhibition of ASFV replication. For the immune sera, the anti-p30, -p54, -pE120R, -pH124R, and -pE184L antisera at 42 dpv also showed great viral inhibition, with marked reduction in fluorescence intensity (Figure 3A and Figure S4). Moreover, the detection of Gluc activity in cell supernatants also showed consistent results (Figure 3B). In summary, in addition to anti-CD2v antibodies, antibodies against p30, p54, pE120R, pH124R, and pE184L can significantly inhibit the replication of ASFV in PAMs.

CD2v binds to the CD2 receptor on the surface of RBCs to promote ASFV proliferation in the host [14]. Despite the absence of viral inhibitory effect in anti-CD2v antisera, the least rosettes of PAMs were observed after incubating with CD2v antisera of 42 dpv (Figure 3C). To ensure the reliability of the results, we counted six fields of view per sample. The number of rosettes of PAMs incubated with antiserum 42 dpv was significantly lower than that of antiserum 0 dpv and SPF pig sera (Figure 3D). Based on the above results, all six antigens, p30, p54, pE120R, pH124R, pE184L, and CD2v, can be used as potential antigen candidates.

### 3.4. Construction and Characterization of Six Nanoparticles Displaying ASFV Antigen Candidates

**Figure 4:**
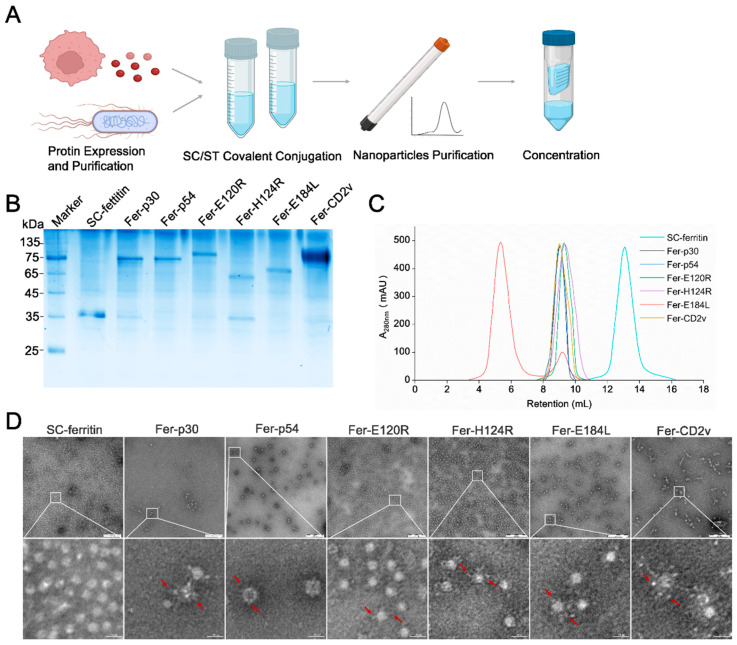
Construction and characterization of six nanoparticles displaying ASFV antigen candidates. (**A**) Procedure of nanoparticle construction. (**B**) Validation for the conjugation of SC-ferritin and monomeric antigens. Coomassie Brilliant Blue staining of SC-ferritin, p30-ferritin (Fer-p30), p54-ferritin (Fer-p54), pE120R-ferritin (Fer-E120R), H124R-ferritin (Fer-H124R), pE184L-ferritin (Fer-E184L), and CD2v-ferritin (Fer-CD2v). (**C**) Self-assembly analysis for the six nanoparticles. The conjugated nanoparticles Fer-p30, Fer-p54, Fer-E120R, Fer-H124R, Fer-E184L, and Fer-CD2v were characterized by SEC. (**D**) Observation of the six nanoparticles by transmission electron microscope (TEM). The images showed the shape of nanoparticles under a 200 nm scale. The white frame is magnified 100 times after the field of view. The red arrow indicates the antigens displayed on the ferritin nanoparticles’ surface.

Purified SC-ferritin was incubated with the antigens. The six antigen-conjugated nanoparticles Fer-p30, Fer-p54, Fer-E120R, Fer-H124R, Fer-E184L, and Fer-CD2v were further purified by SEC and concentrated (Figure 4A). SC and ST form intermolecular isopeptide bonds that irreversibly conjugate ferritin and antigen subunits. The conjugation of the antigen and ferritin was verified by SDS-PAGE and Coomassie Brilliant Blue staining. The results showed that the molecular weight of the nanoparticles coupled with the antigens increased by approximately 35 kDa (molecular weight of SC-ferritin) compared to the weight of the corresponding antigen monomer, indicating that ferritins and the antigens are stably bound by isopeptide bonds (Figure 4B).

Proteins with larger particle sizes were eluted earlier in SEC, enabling the separation of differently sized proteins. While the highest elution peak of ferritin nanoparticles was 13.1 mL in SEC, that of Fer-p30, Fer-p54, Fer-E120R, Fer-H124R, Fer-E184L, and Fer-CD2v eluted at 9.1, 9.0, 9.2, 9.3, 5.3, and 8.9 mL, respectively, which were significantly earlier than that of ferritin nanoparticles alone (Figure 4C). The SEC results implied that the six antigens were successfully coupled with ferritin and had no effect on the self-assembly of ferritin nanoparticles. More details on the nanoparticles can be observed using TEM. Under a field of view of 200 nm scale, six antigen-conjugated nanoparticles exhibited a spherical structure with a particle size of approximately 20 nm. After amplifying the field of vision by 100 times, each kind of antigen, like the “tentacle” could be observed on the ferritin nanoparticles’ surface, while the ferritin nanoparticles’ alone surface has no such phenomenon (Figure 4D). The images of TEM are the most direct evidence to prove the successful construction of six nanoparticles.

### 3.5. Robust Humoral Immune Response Induced After Immunization with Antigen Monomers or Antigen-Conjugated Nanoparticles in Pigs

**Figure 5:**
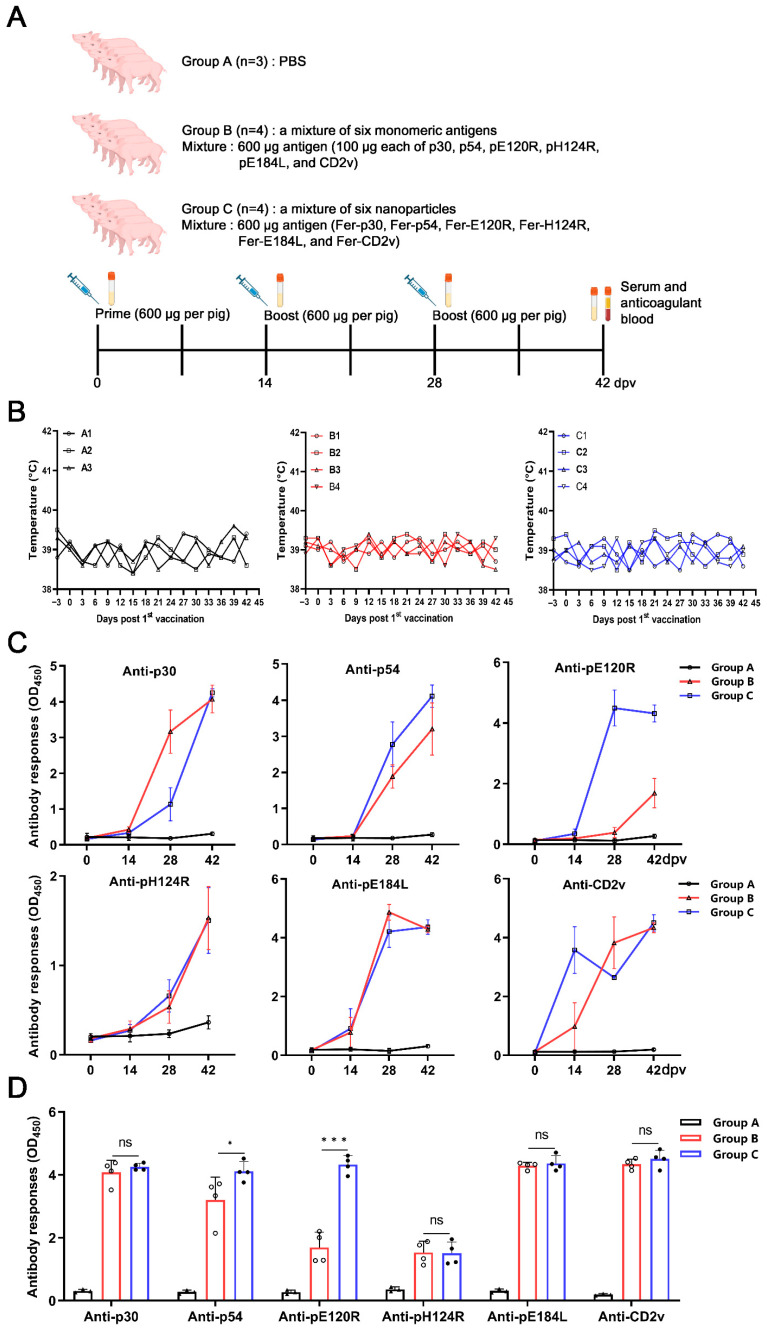
Robust humoral immune response induced after immunization with antigen monomers or antigen-conjugated nanoparticles in pigs. (**A**) Schematic diagram of pig vaccination. Group A received PBS as a negative control (A1, A2, and A3; *n* = 3). Group B was vaccinated with a mixture of six monomer antigens (B1, B2, B3, and B4; *n* = 4). Group C received a mixture of six nanoparticle-conjugated antigens (C1, C2, C3, and C4; *n* = 4). Each pig was administered with 100 μg of each antigen per immunization, with two booster doses at 2 and 4 weeks post-primary vaccination. Blood was collected at 0, 14, 28, and 42 days post-vaccination (dpv). (**B**) The rectal temperature of each pig in Groups A, B, and C at the indicated time points postvaccination. (**C**) The changes in antibodies in pig immunization. Antigen-specific antibody levels were determined by in-house iELISA for p30, p54, pE120R, pH124R, pE184L, and CD2v at 0, 14, 28, and 42 dpv in all pigs. (**D**) Integrated analysis of antibody levels after immunization. Each antigen-specific antibody was determined by in-house iELISA at 42 dpv for three groups. The results were expressed as OD_450nm_. The data were analyzed using the two-way ANOVA (**C**) or one-way ANOVA (**D**), bars represent the means ± SD; ns, not significant (*p* > 0.05); *, *p* < 0.05; ***, *p* < 0.001.

To evaluate the immunogenicity of the antigen combination, we immunized Group B pigs with a mixture of six antigen monomers and Group C pigs with a mixture of six antigen-conjugated nanoparticles, whereas Group A received PBS as a negative control (Figure 5A).

The rectal temperatures of all the pigs were normal during the immunization period (Figure 5B). No significant differences were found in the inflammatory-related cytokines TNF-*α* and IFN-*α* concentration of the sera from the three groups of pigs at 14 dpv (Figure S5A,B). Antibodies against the six antigens were assessed individually during the immunization process. According to the iELISA results, the monomeric antigen group exhibited higher anti-p30 antibody levels at 28 dpv than the antigen-conjugated nanoparticle group, whereas anti-p54 antibody levels were lower. For anti-pE120R antibodies, antigen-conjugated nanoparticles induced significantly higher levels than monomeric antigens at both 28 and 42 dpv. No significant differences were observed in the anti-pH124R and anti-pE184L antibody levels between Group B and Group C during the immunization period. Anti-CD2v antibody titers in the nanoparticle group were significantly higher than those in the monomeric group at 14 dpv, lower at 28 dpv, and comparable at 42 dpv (Figure 5C).

Integrated analysis of antibody levels after immunization revealed no significant differences between the nanoparticle and monomeric groups for anti-p30, -pH124R, -pE184L, and -CD2v antibodies under the current experimental conditions. However, the anti-pE120R and anti-p54 antibody levels were significantly higher in the nanoparticle group (Figure 5D). These results demonstrated that both the combination of monomeric antigens and antigen-conjugated nanoparticles elicited robust humoral immune responses.

### 3.6. Enhanced Virus-Inhibitory Activity of Antibodies Following Immunization with 

#### Antigen-Conjugated Nanoparticles Compared with Antigen Monomers

**Figure 6:**
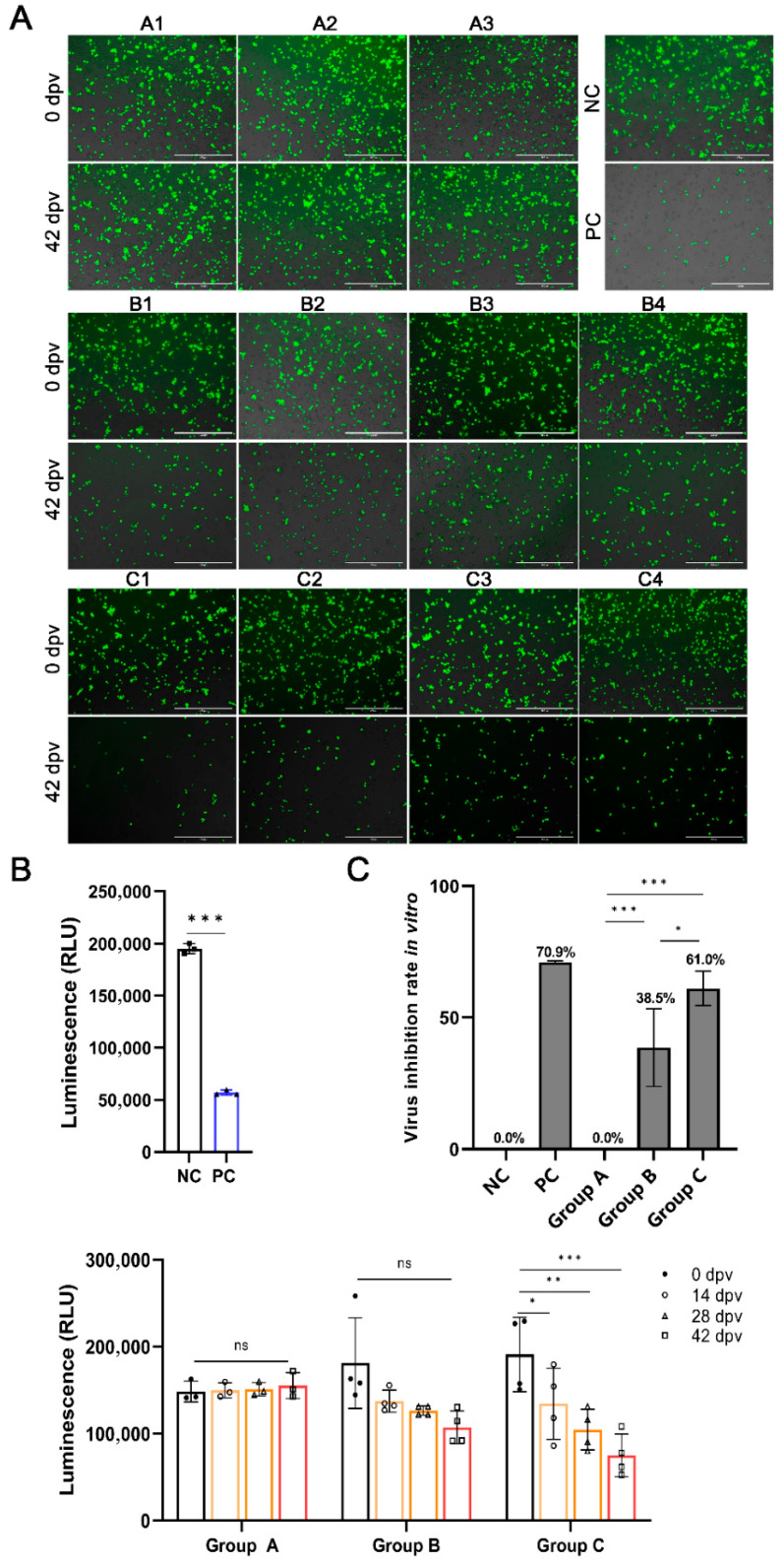
Enhanced virus-inhibitory activity of antibodies following immunization with antigen-conjugated nanoparticles compared with antigen monomers. (**A**) Fluorescence observation of the inhibitory effect. After treatment with serum collected at 0 or 42 dpv from each pig, PAMs infected with rASFV-Gluc/EGFP were observed via fluorescence microscopy at 72 h post-infection (hpi). (**B**) Gluc activities analysis for inhibition. At 72 hpi, the PAMs culture supernatants were collected to detect Gluc activities, and the inhibitory activity of antibodies in the sera of all pigs was analyzed. (**C**) Quantitative analysis of the inhibitory activity of antibodies. The Gluc activities in the 42 dpv sera were compared to those in the 0 dpv sera to calculate the inhibition rate, thereby assessing the extent to which antibodies in the sera of three groups of pigs inhibit ASFV. SPF pig sera were the negative control (NC), and the sera of convalescent pigs were used as the positive control (PC). Group A: pigs injected with PBS (A1, A2, and A3; *n* = 3); Group B: pigs vaccinated with the mixture of six monomer antigens (B1, B2, B3, and B4; *n* = 4); Group C: pigs vaccinated with six antigen-conjugated nanoparticles (C1, C2, C3, and C4; *n* = 4). The data were analyzed using the one-way ANOVA, bars represent the means ± SD; ns, not significant (*p* > 0.05); *, *p* < 0.05; **, *p* < 0.01; ***, *p* < 0.001.

To further assess the ASFV inhibitory activity of antibodies in vitro, sera collected at 0, 14, 28, and 42 dpv were analyzed. Both the monomeric antigen and nanoparticle groups showed reduced fluorescence signals in PAMs after serum treatment at 42 dpv compared with the 0 dpv serum, with a more pronounced reduction in the nanoparticle group. No changes were observed in the PBS group (Figure 6A). Similarly, Gluc activity assays demonstrated enhanced virus inhibition over successive immunizations in the monomeric antigen and nanoparticle groups (8 pigs), while the PBS group (3 pigs) showed no inhibition throughout the experiment (Figure 6B). To compare the degree of inhibition of the monomeric antigen and nanoparticle groups, the virus inhibition rates were calculated. The PBS group showed no inhibition. The inhibition rate of the nanoparticle group (*n* = 4) was as high as 61.0%, which was significantly higher than that of the monomeric antigen group (38.5%; *n* = 4) and close to 70.9% of the positive control (sera of convalescent pigs) (Figure 6C).

### 3.7. Superior Cellular Immune Responses with Antigen-Conjugated Nanoparticle Cocktail Immunization Versus Antigen Monomer Cocktail

**Figure 7:**
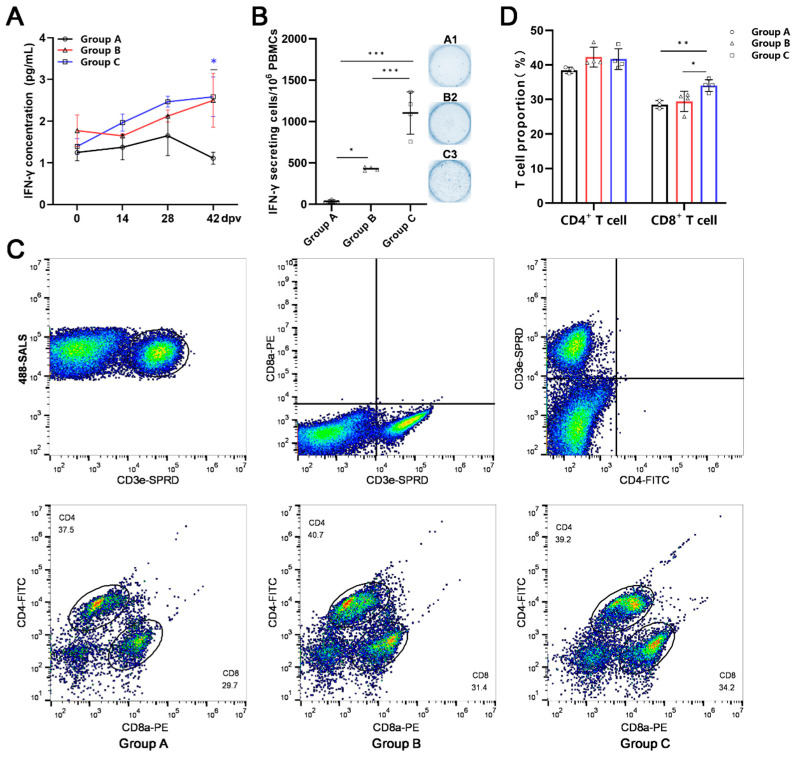
Superior cellular immune responses with antigen-conjugated nanoparticle cocktail immunization versus antigen monomer cocktail. (**A**) Change in serum IFN-*γ* in pig immunization. The serum IFN-*γ* concentrations of the three groups of pigs at 0, 14, 28, and 42 dpv were measured using a commercial kit. (**B**) The detection of ASFV-specific IFN-*γ*-secreting cells. The response of different groups after ASFV stimulation in vitro, IFN-*γ* spot-forming cells, and statistical analysis. Images were acquired and quantified using an ELISpot reader. (**C**) Percentage of CD4^+^ or CD8^+^ T cells. PBMCs were detected by flow cytometry after binding to CD3, CD4, and CD8 specific antibodies with dyes. Group A: pigs injected with PBS (A1, A2, and A3; *n* = 3); Group B: pigs vaccinated with the mixture of six monomer antigens (B1, B2, B3, and B4; *n* = 4); Group C: pigs vaccinated with six antigen-conjugated nanoparticles (C1, C2, C3, and C4; *n* = 4). The data were analyzed using the two-way ANOVA (**A**) or one-way ANOVA (**B**,**D**), bars represent the means ± SD; ns, not significant (*p* > 0.05); *, *p* < 0.05; **, *p* < 0.01; ***, *p* < 0.001.

IFN-*γ* is the core regulatory factor of the cellular immune response. During the immunization period, both the monomeric antigen and nanoparticle groups exhibited upward trends in serum IFN-*γ* concentrations, whereas the PBS group showed no increase. Statistical analysis revealed no significant difference between the monomeric antigen (*n* = 4) and nanoparticle groups (*n* = 4), whereas the nanoparticle group exhibited significantly higher IFN-*γ* levels than the PBS group (*n* = 3) at 42 dpv (Figure 7A). Sera of one pig in the PBS group and one pig in the monomeric antigen group were excluded owing to hemolysis.

To confirm that the IFN-*γ*-secreting effector cells were targeted to ASFV, PBMCs isolated at 42 dpv were stimulated with ASFV-WT and analyzed using ELISpot. The number of IFN-*γ*-secreting PBMCs in the nanoparticle group (*n* = 4) was significantly higher than that in the monomeric antigen group (*n* = 4), with the monomeric antigen group (*n* = 4) surpassing the PBS group (Figure 7B). CD4^+^ and CD8^+^ T cells determine the number of ASFV-specific effector cells. Flow cytometry revealed elevated proportions of CD4^+^ and CD8^+^ T cells in the monomeric antigen (*n* = 4) and nanoparticle groups (*n* = 4). While the proportion of CD4^+^ T cells was comparable between the monomeric antigen (*n* = 4) and nanoparticle groups (*n* = 4), the proportion of CD8^+^ T cells was significantly higher in the nanoparticle group (*n* = 4), which was consistent with the ELISpot results (Figure 7C,D). In conclusion, the combination of antigens induces cellular immunity, and the combination of antigens delivered by nanoparticles can significantly enhance the level of cellular immunity compared to the antigen monomer, particularly in terms of the number of ASFV-specific IFN-*γ*-secreting effector cells.

## 4. Discussion

To screen ASFV antigens, this study evaluated six potential antigen candidates in pigs, including pE120R, pH124R, and pE184L—proteins that have not been previously investigated in ASFV. In Experiment 1, pE120R and pE184L elicited antibody levels comparable to those induced by the well-characterized antigen candidates p30, p54, and CD2v, and antibodies against pH124R demonstrated superior virus inhibitory activity compared to other antigens. Moreover, five of the six candidate antigens we selected (p30, p54, pE120R, pE184L, and CD2v) correspond to the highly immunogenic antigens identified in the latest proteomic study. When delivered using ferritin nanoparticles, these six antigens elicited antibodies that demonstrated an in vitro inhibitory effect on ASFV comparable to that of antibodies from convalescent pig sera, alongside a robust cellular immune response.

We selected ferritin nanoparticles as the delivery platform for two reasons. First, subunit vaccines for ASF often suffer from limited immunogenicity. While our antigen screening aimed to ensure broad and potent humoral immunity, there remained concern regarding the activation of cellular responses. Ferritin nanoparticles have been shown to enhance antigen stability and promote accumulation in lymph nodes, thereby providing sustained stimulation to germinal center B cells, which drives higher levels of somatic hypermutation and induces elevated titers of neutralizing antibodies [38]. Furthermore, their ability to facilitate cross-presentation in antigen-presenting cells (APCs) favors a Th1-biased immune response, enabling more efficient activation of cellular immunity [39,40]. In our previous research on classical swine fever virus (CSFV), ferritin nanoparticle delivery of the protective antigen E2 induced a T-cell response nearly 500 times stronger than that elicited by a conventional subunit vaccine, along with significantly higher neutralizing antibody titers [32]. These results support ferritin nanoparticles as a well-validated delivery system suitable for subsequent experimental applications. Second, the translational potential and safety of ferritin nanoparticles are underscored by the advancement of several ferritin-based influenza vaccines and one SARS-CoV-2 vaccine into Phase I clinical trials, confirming their safety and stability profiles [41,42,43]. Given these advantages, ferritin nanoparticles were employed as the antigen delivery vector in Experiment 2.

The screening of antigen candidates is a critical step in the development of ASF subunit vaccines. Passive immunization of pigs with the immunoglobulin derived from pigs that survived challenge with the ASFV E75 strain conferred 85% protection against homologous challenge, indicating that antibodies targeting specific viral antigens contribute substantially to immunity and are closely associated with protection against ASFV [44]. However, not all antibody responses are beneficial; we previously reported that anti-A137R antibodies enhance ASFV infection in PAMs via antibody-dependent enhancement and accelerate clinical symptom onset in infected pigs [34,45]. Therefore, in this study, in addition to predicting and validating antigen immunogenicity in vivo, we included the in vitro virus inhibitory activity of antigen-specific antibodies as a key criterion for candidate selection. Notably, none of the six antigens evaluated here promoted ASFV infection (Figure 3B). The inhibitory effects of anti-p30 and anti-p54 antibodies observed in our assays are consistent with previous reports [16]. Significantly, antibodies against pE120R, pH124R, and pE184L—identified here for the first time as inhibitors—expand the repertoire of antigen candidates for ASF vaccine design. We also noted that not all porcine sera raised against the same antigen exhibited virus inhibitory effects, which may reflect inter-individual variation in epitope recognition or differences in the immunodominance hierarchy. In Experiment 1, several pigs immunized with pH124R, pE184L, or CD2v became infected with *Glaesserella parasuis*. Despite preliminary treatment, four pigs were euthanized between 9 and 12 dpv. This reduction in sample size limited the analysis of antibody responses. Due to the decrease in sample size in certain groups, we opted to present the data by repeatedly measuring each individual’s serum ability to inhibit ASFV infection and comparing it with their pre-immunization serum (Figure 3B) using rASFV-Gluc/EGFP infection experiment. CD2v, an outer envelope protein exploited by ASFV to adsorb RBCs and facilitate systemic spread [14], was further evaluated to confirm hemadsorption inhibitory activity observed with CD2v antisera (Figure 3C). Sera from the mixed-antigen monomer group (Group B, *n* = 4), which contained anti-CD2v antibodies, also exhibited hemadsorption inhibitory activity across all four pigs, supporting the relevance of CD2v as an antigen candidate (Figure S6). Furthermore, we compared antigen-specific antibody titers at 42 dpv from single-antigen immunizations (Experiment 1) and the six-antigen monomer mixture (Group B, Experiment 2). Antibody levels against p30, p54, pE184L, and CD2v were not significantly affected in the mixture, suggesting no antigenic competition among these four proteins. In contrast, pE120R showed evidence of antigenic competition: in single immunization, OD_450nm_ values were 4.352, 0.330, and 2.172 (Figure 2D), whereas in the mixed-monomer group they dropped to 1.274, 1.282, 2.005, and 2.200 (Figure 5B). Notably, this suppression was reversed by ferritin nanoparticle delivery, with the nanoparticle-mixture group achieving OD_450nm_ values of 4.454, 4.612, 4.269, and 3.959 (Figure 5B)—indicating that nanoparticle presentation may overcome antigenic competition by enhancing antigen stability, thereby significantly potentiating the humoral immune response. In the case of pH124R, its generally low immunogenicity may explain why the delivery vector did not notably enhance it. These findings provide two key implications for future vaccine design: (1) Antigen screening. The evaluation of structural stability and competitive performance within multi-antigen formulations may be included for antigen selection. (2) Delivery platform. The delivery system critically determines vaccine outcome, requiring the suitable systems to overcome antigen competition.

Ferritin nanoparticle delivery significantly enhanced the overall immunogenic efficacy of a multi-antigen candidate vaccine against ASFV. While adenoviral vectors and liposomes have been reported for multi-antigen delivery in ASF vaccine development [46,47], ferritin nanoparticles have primarily been employed to display single antigen or tandem T-cell epitopes in prior studies [48,49], with limited data available on their use for multi-antigen immunization in pigs. The enhancement in antigen immunogenicity of ferritin is well recognized. However, in this study, we found that not all ASFV antigens delivered via ferritin demonstrated significantly higher antibody titers compared to the monomeric antigen immunization. Notably, the viral inhibitory effect of sera from the nanoparticle-immunized group increased progressively throughout the vaccination course. Specifically, the viral inhibition rate of sera at 42 dpv rose from 38.5% with mixed monomeric antigens to 61.0% with nanoparticle-formulated antigens (Figure 6B,C). We propose two non-mutually exclusive explanations for this enhancement. First, nanoparticle presentation may enhance the presentation of antibodies targeting key epitopes of six antigens, increasing their quantity or affinity—consistent with our earlier observations in a CSFV ferritin nanoparticle vaccine [32]. Second, p54 and pE120R exhibit lower predicted stability compared to the other four antigens. We performed protein structure prediction (AlphaFold3, https://alphafoldserver.com/, accessed on 8 January 2026) and B-cell epitope mapping (IEDB Analysis Resource, https://tools.iedb.org/bcell/, accessed on 8 January 2026) to assess epitope accessibility. Although no distinct epitopes were uniquely localized within the antigens (Figure S7A), the secondary structures of p54 and pE120R were markedly different, characterized by substantially higher random coil contents (80.0% and 69.0%, respectively) than the other four antigens (Figure S7B). We therefore propose that conjugation to ferritin nanoparticles enhances the structural stability of p54 and pE120R, thereby promoting more efficient B-cell activation and elevated antibody production in Group C (Experiment 2). This structural instability may also account for the fluctuating antibody titers observed in the p54- and pE120R-immunized pigs in Experiment 1. This mechanism, where nanoparticle attachment improves antigen stability and immunogenicity, has been documented in multiple vaccine studies targeting pathogens such as rabies virus, SARS-CoV-2, and PRRSV [27,50,51]. Interestingly, convalescent pig sera used as a positive control in the viral inhibition assay showed a 70.9% inhibition rate rather than complete neutralization. We speculate that this partial inhibition may relate to the structural diversity of ASFV virions. Both the external envelope of extracellular virions and the membrane of ApoBD-containing virions incorporate various viral antigens and host molecules, which may help shield the virus from antibody binding. The impact of ASFV particle heterogeneity on antibody-mediated protection of ASF vaccines merits further investigation [52]. The gap between the 38.5% inhibition rate induced by mixed monomer immunization and the 70.9% rate observed with the sera of convalescent pigs also needs to be investigated. Furthermore, multiple cellular immunity readouts were markedly enhanced in the nanoparticle group. We attribute this to improved antigen uptake, processing, and presentation by APCs facilitated by ferritin nanoparticles, leading to elevated IFN-*γ* secretion, a strengthened Th1-biased CD4^+^ T cell response, enhanced CD8^+^ T cell activation and differentiation, and an expanded pool of ASFV-specific CD8^+^ T cells. Collectively, these data demonstrate that ferritin nanoparticles serve as an effective antigen delivery platform for multi-antigen ASF subunit vaccines, significantly enhancing both the humoral and cellular arms of the immune response.

Immunological correlates of protective immunity against ASFV are not fully defined. To date, there is no direct evidence that the virus inhibitory activity of antibodies observed in vitro translates into protective immunity in vivo. It is hypothesized that antibody-mediated protection may operate through mechanisms such as antibody-dependent complement deposition (ADCD) or antibody-dependent cellular cytotoxicity (ADCC) [53]. In support of this, anti-p30 antibodies have been shown to trigger NK cell-mediated ADCC, and the anti-pE120R antibodies examined in this study also demonstrated ADCC activity [54,55]. Establishing a clearer relationship between in vitro neutralization or functional assays and in vivo protection will greatly facilitate the rational and efficient development of effective ASF vaccines.

The key to the development of ASF subunit vaccines is the discovery and strategic combination of ASFV protective antigen candidates and the selection of delivery systems. While this study has identified six candidate antigens and enhanced their immunogenicity using ferritin nanoparticles, further work remains necessary: (i) efficiency evaluation in pigs; (ii) mechanistic studies on antibody-mediated inhibition (e.g., ADCD and ADCC); (iii) identification of critical epitopes and novel antigen candidates. These steps are expected to advance the development of safe, effective, and economical ASF vaccines.

## 5. Conclusions

This preclinical study demonstrates that ferritin nanoparticle delivery can enhance both humoral and cellular immune responses against ASFV in a screened multi-antigen formulation. The six antigen candidates—p30, p54, pE120R, pH124R, pE184L, and CD2v—elicited functional antibodies capable of inhibiting viral replication or hemadsorption. Nanoparticle presentation significantly improved antibody-mediated virus inhibition and promoted strong CD8^+^ T cell and IFN-*γ* responses. These findings, derived from antigen screening and immunogenicity evaluation, provide a promising nanoparticle-based strategy for the future development of effective ASF subunit vaccines.

## Figures and Tables

**Table 1:** The primers for the construction of the plasmids.

Primers	Sequences (5′-3′)
Ag-F	CATATGGAGCTCGGTACCCTCGAG
Ag-R	CACCTCCGCCCGAGCCTCCGCCACC
ferritin-F	GGTGGCGGAGGCTCGGGCGGAGGTGGGTCGGGTGGCGGCGGATC
ferritin-R	GCAGAGATTACCTATCTAGATCAGTGGTGGTGGTGATGATGTGCGCTTTTTTCAAATTGAGGATGGCTCCAGCTACCACGCGGAACCAGACCACCAGATTTCCTGCTTTTAGCGATCCCTT
ST-F	CATGGTGGATGCGTACAAACCGACCAAGGGTGGTCTGGTTCCGCGT
ST-R	TGTACGCATCCACCATGACGATGTGCGCTGATCCGCCGCCACCCGA

## Data Availability

The data that support the findings of this study in this manuscript are available at ScienceDB (https://doi.org/10.57760/sciencedb.27173, accessed on 8 January 2026).

## Supplementary Materials

The following supporting information can be downloaded at: https://www.mdpi.com/article/10.3390/vaccines14010093/s1, Figure S1: Construction of antigen-expressing strains or a CHO cell line; Figure S2: Purification of antigen candidates; Figure S3: IFA detection for the sera at 42 dpv of all pigs; Figure S4: Fluorescence observation for inhibition of ASFV replication by antibodies; Figure S5: The concentrations of inflammatory-related TNF-*α* (A) and IFN-*α* (B) in the serum of pigs in Groups A, B, and C at 14 days post-vaccination; Figure S6: Hemadsorption inhibition of Group B antisera; Figure S7: Structural prediction and analysis of six antigen candidates; Table S1: The cut-off values of the six in-house iELISAs.

## Abbreviations

The following abbreviations are used in this manuscript:

ASF	African swine fever
ASFV	African swine fever virus
PAMs	Primary porcine alveolar macrophages
LAVs	Live attenuated vaccines
SPF	Specific pathogen-free
FBS	Fetal bovine serum
ASFV-WT	ASFV HLJ/18 strain
ST	SpyTag
SC-ferritin	SpyCatcher-ferritin
Tris-HCl	Hydroxymethyl aminomethane hydrochloride
PBS	Phosphate-buffered saline
HEPES	4-(2-Hydroxyethyl) piperazine-1-ethanesulfonic acid
HRP	Horseradish peroxidase
IFA	Indirect immunofluorescence assay
iELISA	Indirect enzyme-linked immunosorbent assay
HADIA	Hemadsorption inhibition assay
RBCs	Red blood cells
SEC	Size-exclusion chromatography
TEM	Transmission electron microscope
dpv	Days post-vaccination
Fer-p30	p30-ferritin
Fer-p54	p54-ferritin
Fer-E120R	pE120R-ferritin
Fer-H124R	pH124R-ferritin
Fer-E184L	pE184L-ferritin
Fer-CD2v	CD2v-ferritin
PBMCs	Peripheral blood mononuclear cells
ELISA	Enzyme-linked immunosorbent assay
ELIspot	Enzyme-linked immunosorbent spot
APCs	Antigen-presenting cells
CSFV	Classical swine fever virus
ADCD	Antibody-dependent complement deposition
ADCC	Antibody-dependent cellular cytotoxicity
