# Supplementary material for: A Pool of Ferritin Nanoparticles Delivering Six Proteins of African Swine Fever Virus Induces Robust Humoral and Cellular Immune Responses in Pigs
Source: Vaccines (Basel). 2026 Jan 19;14(1):93. doi: 10.3390/vaccines14010093 (PMC12846370; doi:10.3390/vaccines14010093)
Supplement: Supplementary file 1 [file vaccines-14-00093-s001.zip › vaccines-4073049-supplementary.pdf]

Table 1. The cut-off values of the six in-house iELISAs.

Serum	Cut-off value
Anti-p30	0.108 ± 0.130
Anti-p54	0.088 ± 0.076
Anti-pE120R	0.097 ± 0.097
Anti-pH124R	0.095 ± 0.091
Anti-pE184L	0.103 ± 0.095
Anti-CD2v	0.086 ± 0.064

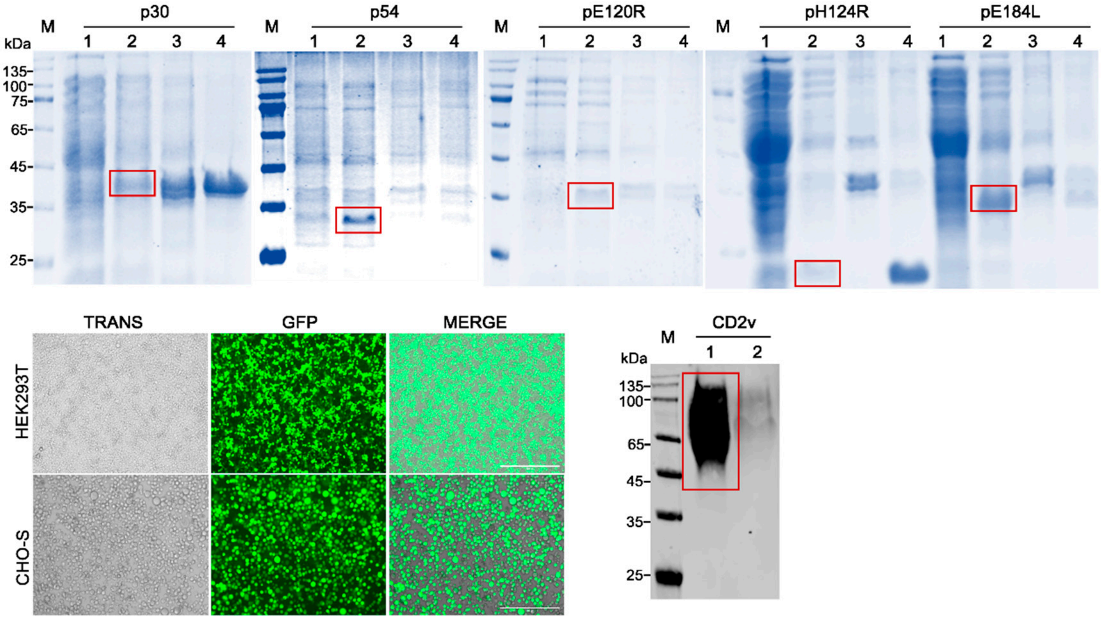

Figure S1. Construction of antigen-expressing strains or a CHO cell line. (A) Verification of prokaryotic expression of antigens. M: Marker; 1: Uninduced supernatant after sonication; 2: Induced supernatant after sonication; 3: Uninduced precipitation after sonication; 4: Induced precipitation after sonication. (B) Construction of CHO-CD2v-ST cell line. (C) Verification of CD2v expression. M: Marker; 1: CHO-CD2v-ST cell culture supernatant; 2: CHO-S-WT cell culture supernatant.

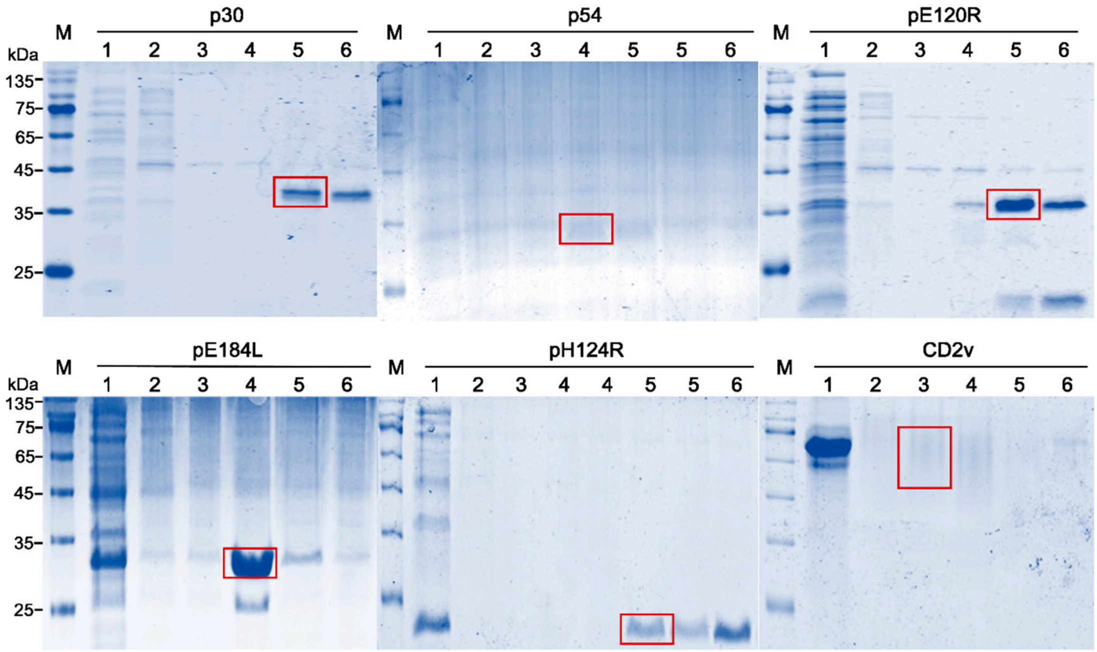

Figure S2. Purification of antigen candidates. M: Marker; 1: Flow-through fluid; 2: 20 mM imidazole; 3: 50 mM imidazole; 4: 100 mM imidazole; 5: 200 mM imidazole; 6: 500 mM imidazole.

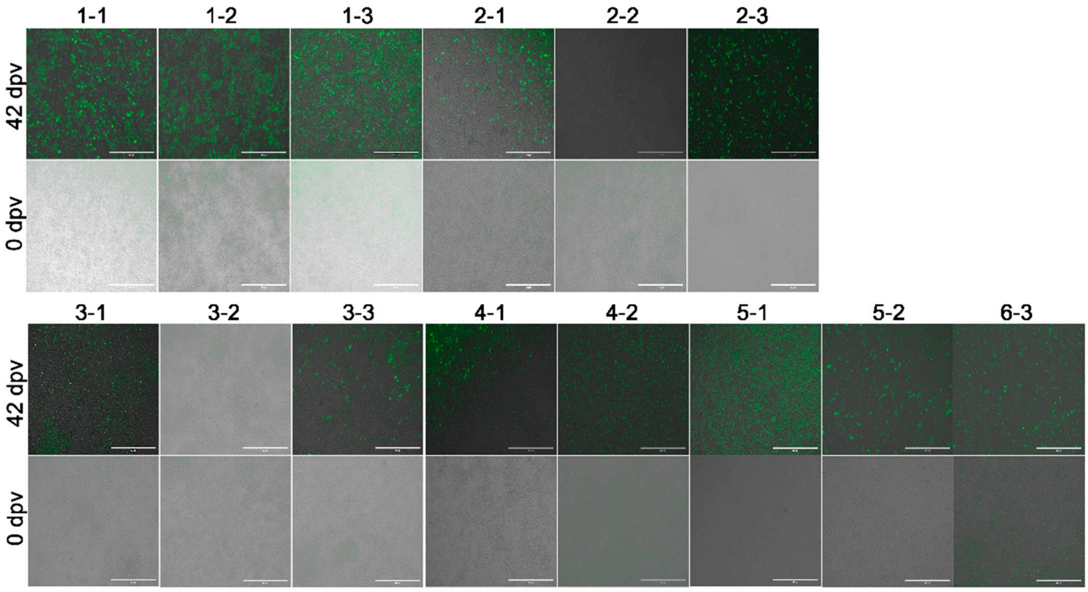

Figure S3. IFA detection for the sera at 42 dpv of all pigs. Group 1: pigs vaccinated with p30 (1-1, 1-2 and 1-3; n=3); Group 2: pigs vaccinated with p54 (2-1, 2-2 and 2-3; n=3); Group 3: pigs vaccinated with pE120R (3-1, 3-2 and 3-3; n=3); Group 4: pigs vaccinated with pH124R (4-1 and 4-2; n=2); Group 5: pigs vaccinated with pE184L (5-1 and 5-2; n=2); Group 6: pigs vaccinated with CD2v (6-3; n=1).

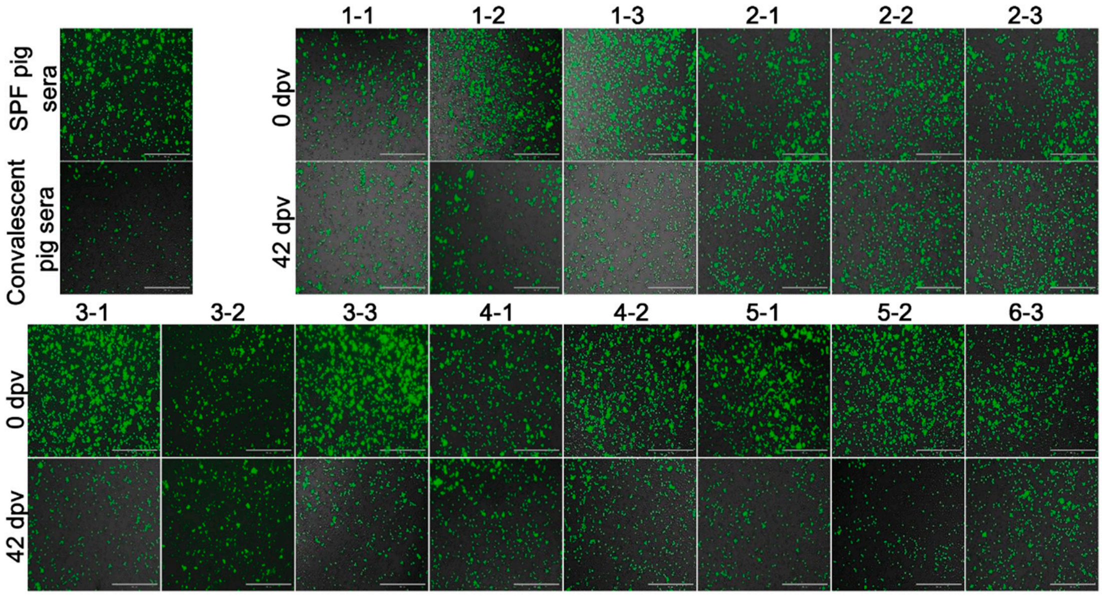

Figure S4. Fluorescence observation for inhibition of ASFV replication by antibodies. Group 1: pigs vaccinated with p30 (1-1, 1-2 and 1-3; n=3); Group 2: pigs vaccinated with p54 (2-1, 2-2 and 2-3; n=3); Group 3: pigs vaccinated with pE120R (3-1, 3-2 and 3-3; n=3); Group 4: pigs vaccinated with pH124R (4-1 and 4-2; n=2); Group 5: pigs vaccinated with pE184L (5-1 and 5-2; n=2); Group 6: pigs vaccinated with CD2v (6-3; n=1). SPF pig sera were the negative control, and the sera of convalescent pigs were used as the positive control.

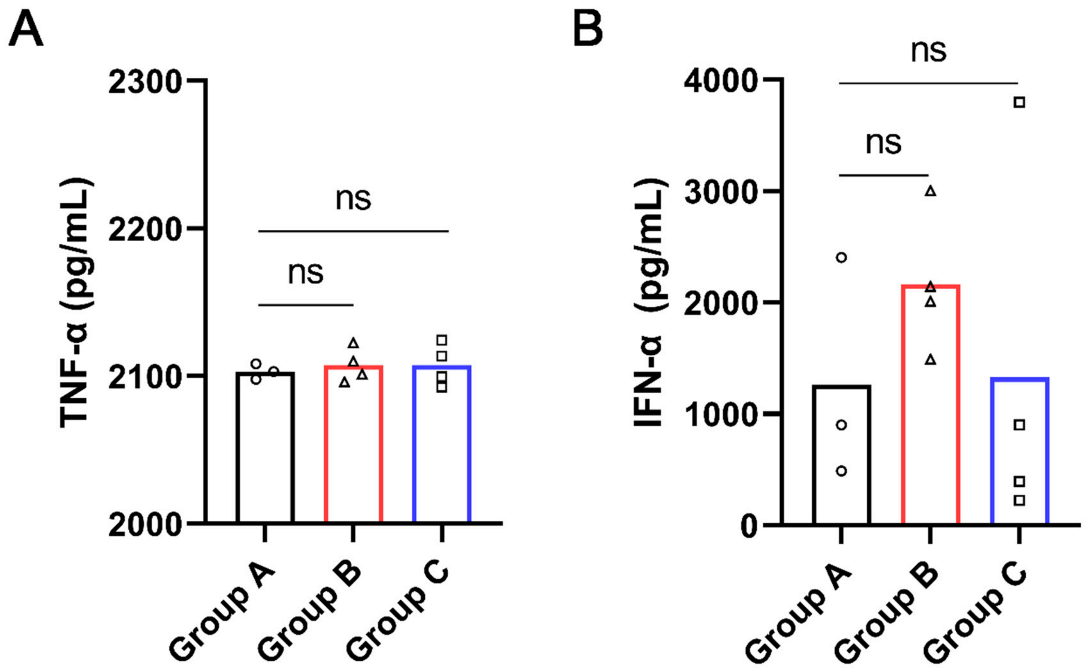

Figure S5. The concentrations of inflammatory-related TNF-*α* (A) and IFN-*α* (B) in the serum of pigs in Groups A, B, and C at 14 days post-vaccination. Group A received PBS as a negative control (A1, A2 and A3; n=3). Group B was vaccinated with a mixture of six monomer antigens (B1, B2, B3, and B4; n=4). Group C received a mixture of six nanoparticle-conjugated antigens (C1, C2, C3, and C4; n=4). The data were analyzed using the one-way ANOVA, bars represent the means ± SD; ns, not significant ($p > 0.05$); *, $p < 0.05$; **, $p < 0.01$; ***, $p < 0.001$.

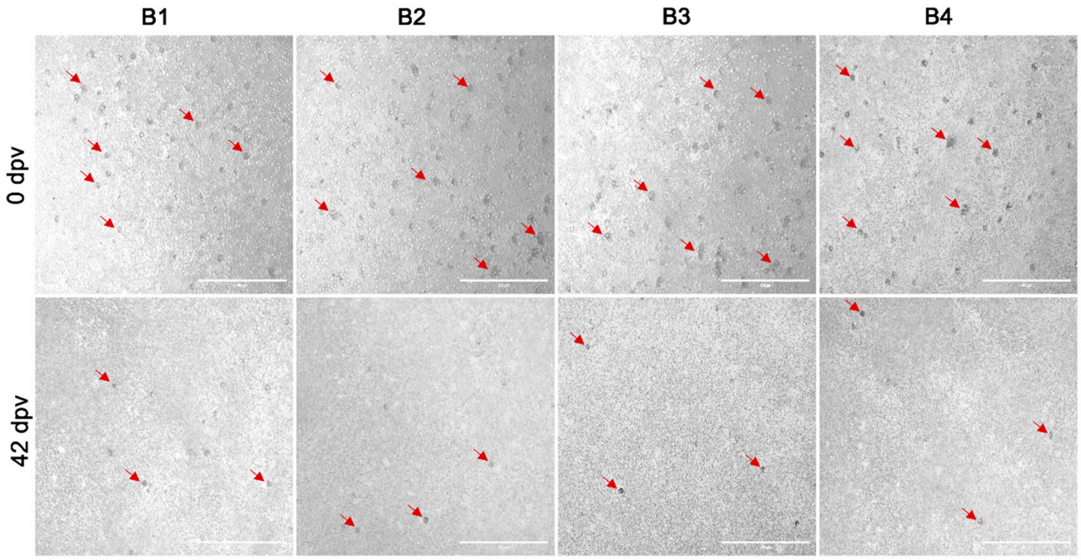

Figure S6. Hemadsorption inhibition of Group B antisera. Four antisera were respectively incubated with ASFV-WT-infected PAMs for 2.5 h. Hemadsorption was observed after adding 30 μL of 1% red blood cells for 36 h. Group B was vaccinated with a mixture of six monomer antigens (B1, B2, B3, and B4; n=4).

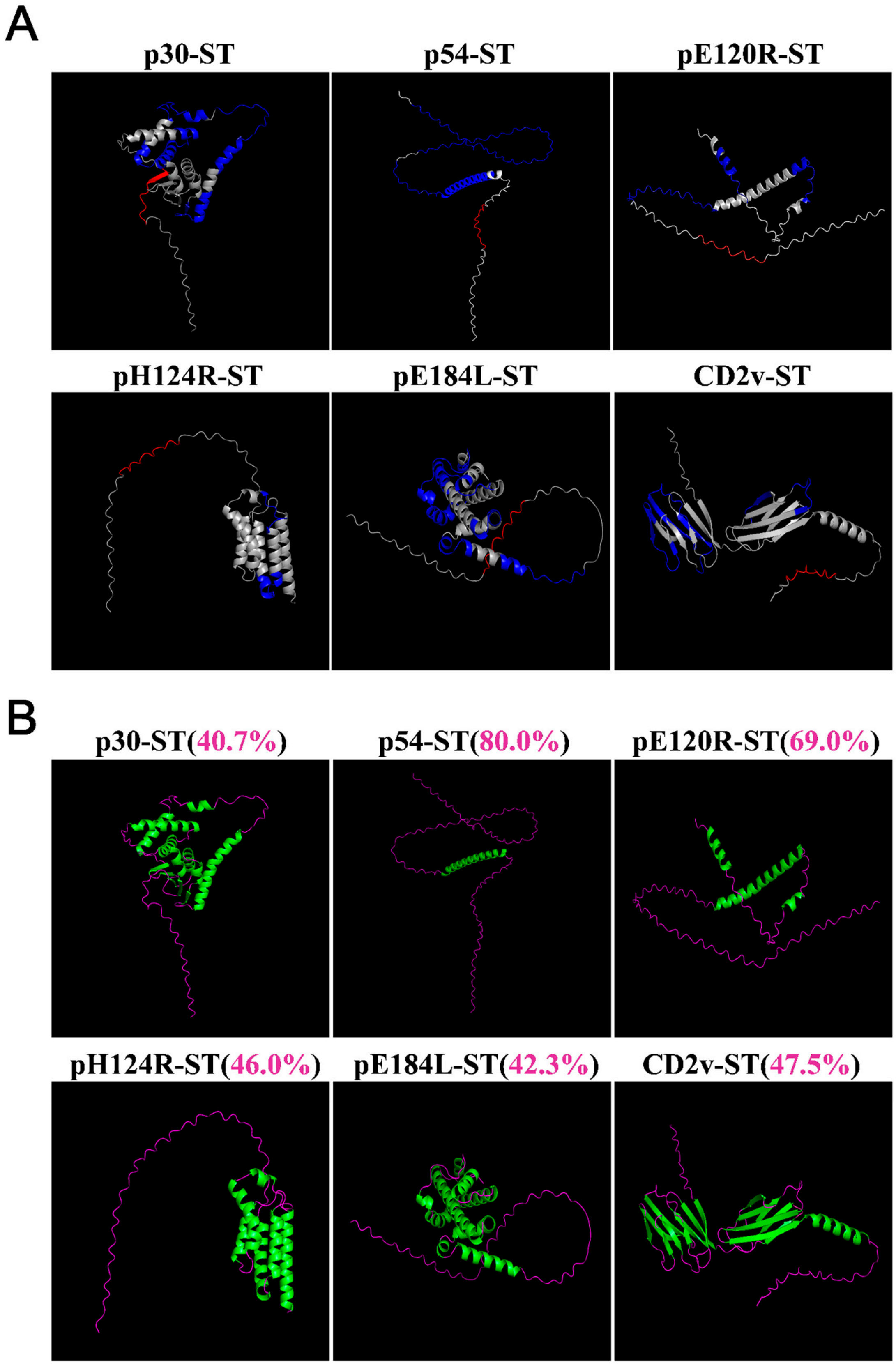

Figure S7. Structural prediction and analysis of six antigen candidates. (A) The accessibility of antigenic epitopes. AlphaFold3 (https://alphafoldserver.com/) was used for protein structure prediction. B-cell epitope mapping was predicted by IEDB Analysis Resource (https://tools.iedb.org/bcell/). B-cell epitopes were highlighted in blue. SpyTag was shown in red. (B) The characteristics of the secondary structure of

antigens. Random coils were colored purple. Beta-sheet and alpha-helix were depicted in green. These in silico structural predictions (e.g., higher random coil content of p54 and pE120R) provide a context for interpreting the differential enhancement of antibody responses by ferritin nanoparticles, as proposed in the Discussion.
